# Effect of sodium fluoride pretreatment on the efficacy of an in‐office bleaching agent: An in vitro study

**DOI:** 10.1002/cre2.113

**Published:** 2018-06-29

**Authors:** Khin Yupar Kyaw, Masayuki Otsuki, Michelle Sunico Segarra, Junji Tagami

**Affiliations:** ^1^ Cariology and Operative Dentistry, Department of Restorative Sciences, Graduate school of Medical and Dental Sciences Tokyo Medical and Dental University Japan; ^2^ Section of Operative Dentistry, College of Dentistry University of the Philippines Philippines

**Keywords:** sodium fluoride, tooth bleaching, tooth color

## Abstract

This in vitro study evaluated the effect of sodium fluoride (NaF) on the bleaching efficacy using an artificial discolored bovine tooth model. Twenty specimens were prepared from bovine teeth by staining with black tea extract and were divided into two groups (*n* = 10). In control group, specimens were immersed in distilled water for 30 min. In NaF group, specimens were applied with 0.2% NaF for 30 min. Then, the specimens in each group were bleached by an in‐office bleaching material (Shofu Hi‐Lite, Shofu, Kyoto, Japan). The CIE L*a*b* values were measured by a dental colorimeter before and after 10 consecutive bleaching treatments, and the color difference (ΔE) was calculated. Brightness and color difference (ΔE) increased in both groups per bleaching cycle. There were no statistical differences in ΔE values between both groups (*p* > 0.05). It was concluded that the application of NaF before bleaching did not interfere with the bleaching effect.

## 1 INTRODUCTION

Tooth bleaching is one of the most conservative and cost‐effective dental treatments and is highly demanded by patients desiring to improve their smile. There are several methods of tooth bleaching; professionally applied in the dental office (in‐office bleaching); dentist‐prescribed patient at‐home use (at‐home bleaching), over‐the‐counter, and other nondental options. The in‐office bleaching is applied in a dental in‐office, and one or several visits are required to achieve satisfactory results. Generally, the active ingredient of in‐office bleaching agents is a high concentration of hydrogen peroxide (ADA Council on Scientific Affairs, 2009). Hydrogen peroxide generates unstable free radicals that attack the organic chromogenic molecules in the tooth substrate and break down these pigments smaller transparent molecules (Sulieman, 2008). One of the most frequent adverse effects of tooth bleaching is postoperative dentin hypersensitivity (ADA Council on Scientific Affairs, 2009). Almost two‐thirds of patients who undergo tooth bleaching experienced transient tooth sensitivity during and after the procedure (Haywood, Leonard, Nelson, & Brunson, 1994).

Prescribing analgesics, application of desensitizing toothpastes, fluorides, and reducing the gel concentration and decreasing the treatment time (for at‐home bleaching) have been suggested to prevent and treat bleaching related postoperative sensitivity (Armênio et al., 2008; Croll, 2003; Giniger, Macdonald, Ziemba, & Felix, 2005; Haywood, 2005; Jorgensen & Carroll, 2002). A recent in vivo study demonstrated that a desensitizing gel containing 5% potassium nitrate and 2% sodium fluoride (NaF) prior to in‐office bleaching reduced the incidence and intensity of hypersensitivity without affecting the bleaching efficacy (Tay, Kose, Loguercio, & Reis, 2009). Some in‐office bleaching products contain NaF.

However, according to manufacturer's instruction of an in‐office bleaching agent (Shofu Hi‐Lite, Shofu, Kyoto, Japan), fluoride‐containing abrasive pastes cannot be used for teeth cleaning prior to the tooth bleaching procedure. No study has addressed the effect of NaF use prior to bleaching on the bleaching efficacy of this particular in‐office bleaching agent.

The purpose of this study was to evaluate the effect of the application of NaF on in‐office bleaching efficacy in vitro. The null hypothesis was that the application of NaF does not interfere with the bleaching effect of Shofu Hi‐Lite.

## 2 MATERIALS AND METHODS

### 2.1 Preparation of discolored tooth specimens

**Figure 1:**
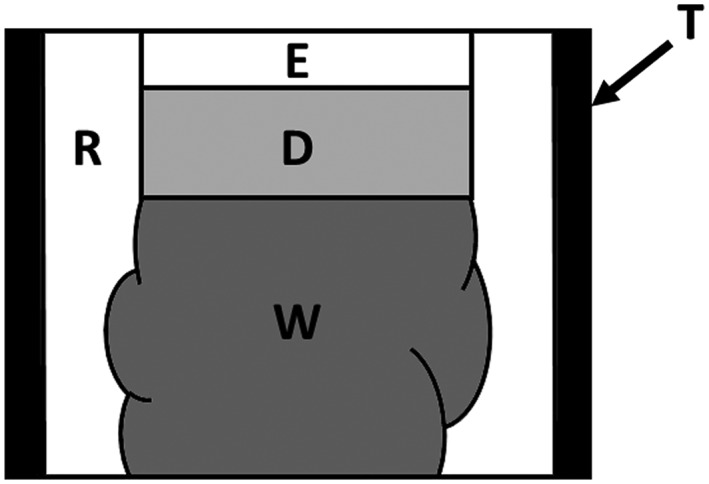
Schematic illustration of an experimental samples, T: cylindrical tube, E: enamel, D: dentin, W: dental wax, R: acrylic resin. Wax was removed after polymerization of acrylic resin for embedding

Extracted bovine incisors kept frozen were used for this study. The teeth were thawed in running tap water and were gently cleaned of soft tissue remnants using a scalpel. The labial surfaces were ground and polished with silicon carbide papers of ascending grits from #180 to #800 (Sankyorikagaku, Saitama, Japan) until enamel of approximately 1 mm in thickness was left. Two specimens of size of approximately 5 mm × 5 mm were obtained from the flattened surface of each tooth using a rotary diamond saw (Mini Lab‐cutter MC‐110, Maruto Instrument, Tokyo, Japan) under copious water. The pulpal dentin of each specimen was covered with dental wax (Utility Wax, GC, Tokyo, Japan), and the specimen was embedded in a cylindrical acrylic tube with 10 mm in height and 10 mm in internal diameter using dental self‐curing acrylic resin (Uni Fast II, GC) as schematically shown in Figure 1. After polymerization of the embedding resin, the wax was removed and the dentin surfaces were irrigated with 5% sodium hypochlorite solution (Wako Pure Chemical, Osaka, Japan) for 1 min to remove organic tissue remnants. The dentin surfaces were then etched with 40% phosphoric acid gel (K‐etchant GEL, Kuraray Noritake Dental, Tokyo, Japan) for 10 sec to open the dentinal tubules to facilitate stain uptake into dentin. The enamel surface of specimens was polished again with #1000 and #1200 grit silicon carbide paper.

The tea solution was extracted by immersing two tea bags (Lipton Yellow Label Teabag, Unilever Japan, Tokyo, Japan) in 50‐ml boiling water for 5 min. The specimens were immersed in the tea extract and stored in an incubator for 7 days at 37°C. The tea solution was changed on the fourth day of the experiment.

After rinsing with tap water and air drying, the color of the enamel surfaces was measured with a dental colorimeter (Shade Eye NCC, Shofu, Kyoto, Japan) and the CIE L*a* b* values were recorded. The color measurement was performed three times and average of obtained data was employed as the representative data. Photographs of the surfaces were also taken with a digital camera. To standardize the color of stained samples, 20 specimens that showed L* values between 50 and 55 were selected for the following experiment. These specimens were randomly assigned to two experimental groups of 10 samples each.

Control group (No pretreatment): The specimens were stored in distilled water for 30 min before the bleaching procedure.

NaF group (Pretreated with NaF solution): The 0.2% NaF solution was prepared and each specimen was immersed in 50 ml of that solution for 30 min before the bleaching procedure.

After the immersion, the color was measured again with the colorimeter and photographs were also taken. Those L*, a*, and b* values were employed as a baseline.

### 2.2 Tooth bleaching

The specimens were bleached with an in‐office bleaching agent (Shofu Hi‐Lite, Shofu) following manufacturer's instructions. The components of this bleaching material are listed in Table 1. One scoop of powder and three drops of liquid were mixed for 30 sec using a spatula until a uniform paste was formed. The paste was applied in 1–2 mm thickness on the enamel surface of specimens. The paste was left on the surface for approximately 6 min and then was irradiated by a halogen light unit with a power intensity of 600 mW/cm^2^ (Optilux 501, Demetrom, Danbury, CT, USA) for 3 min. The paste was left on the surface for another 2 min and was then removed with damp cotton. The specimens were washed thoroughly under tap water, dried gently, and color measurements and photographs were performed. Bleaching treatments and color measurements were repeated 10 times on each specimen.

**Table 1:** Materials and their composition used in this study

Products and manufactures	Composition
Sodium fluoride (NaF) Wako Pure Chemical, Osaka, Japan	Sodium fluoride
Shofu Hi‐Lite Shofu, Kyoto, Japan	Powder Potassium peroxohydrogensulphate, Potassium hydrogensulphate, Manganese sulphate, Hydrated amorphous silica, Poly gantrez Ms‐955, Guinea green dye Liquid 35% hydrogen peroxide

The difference of L*, a*, and b* between the baseline and each bleaching period was expressed as ΔL, Δa, and Δb, respectively. The color difference (ΔE) from the values obtained at the baseline and after each bleaching application period was calculated using the following equation:

$$\mathrm{\Delta E}={\left({\mathrm{\Delta L}}^{2}+{\mathrm{\Delta a}}^{2}+{\mathrm{\Delta b}}^{2}\right)}^{1/2}.$$

### 2.3 Statistical analysis

Two‐way ANOVA followed by Tukey's Honestly Significant Difference (HSD) at 95% level of confidence (SPSS Statistics Ver 21, IBM, Armonk, NY, USA) was done to determine statistically significant differences in ΔE between NaF and control groups as well as among a number of bleaching cycles.

## 3 RESULTS

**Figure 2:**
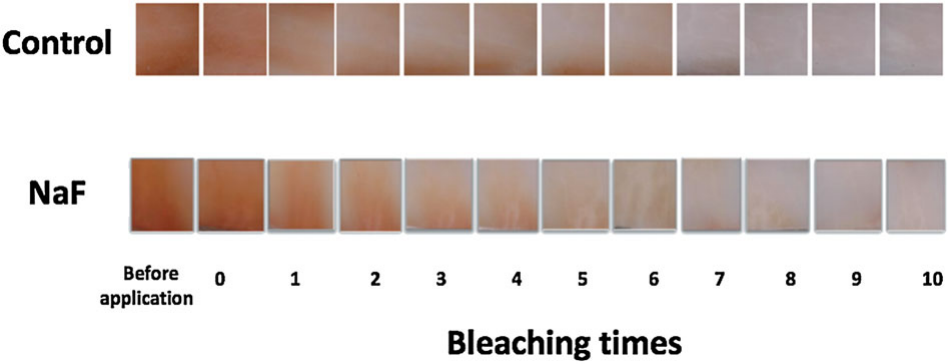
Typical color change at each bleaching step

**Figure 3:**
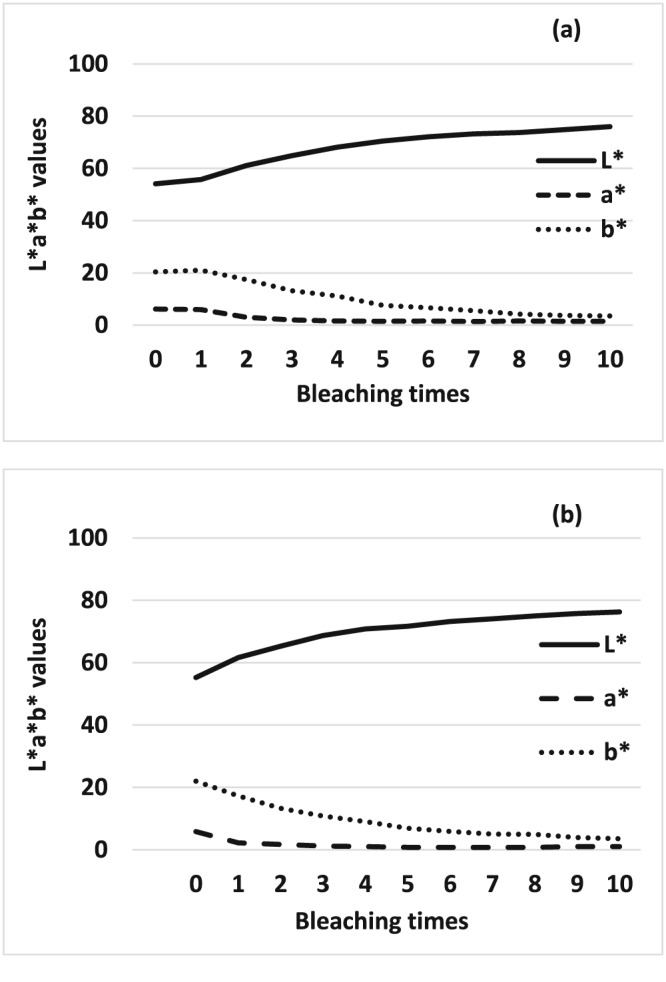
Comparison of changes of the average L*a*b* values of each period. (a) control group and (b) sodium fluoride group

**Figure 4:**
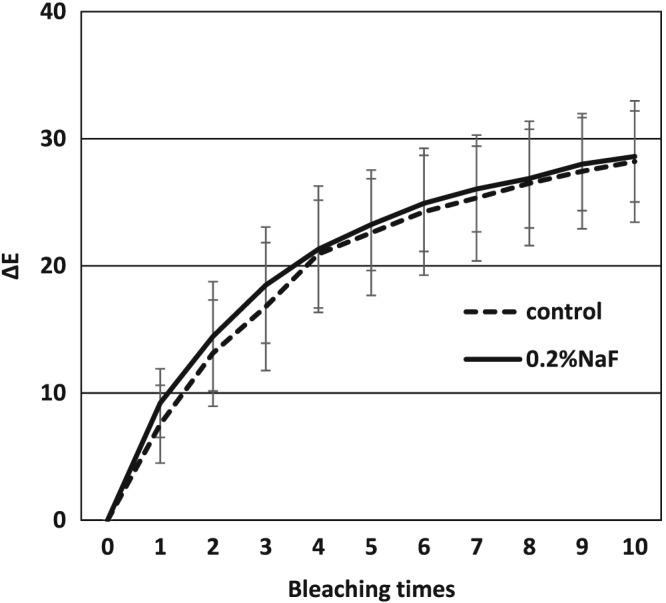
ΔE changes after bleaching between control group and NaF group. No significant difference was observed between the two groups to the figure legend. NaF: sodium fluoride

The typical color changes were shown in Figure 2. The average L* a* b* values of each bleaching treatment were demonstrated in Figure 3. A gradual but remarkable bleaching efficacy was observed after 10 applications of the bleaching agent for both groups as evidenced by increasing L* value and decreasing a* and b* values. The color changes in NaF group was almost similar to that of control group. The values of ΔE were shown in Figure 4. According to increasing the number of bleaching times, ΔE values were also gradually increased in both groups. In turn, two‐way analysis of variance showed that ΔE of 0.2% NaF group was not significantly different with that of the control group (*p* > 0.05).

## 4 DISCUSSION

Tooth sensitivity is a primary and one of the most common negative effect of teeth bleaching and is the main deterrent to a patients' successful completion of the treatment. Postoperative transient mild to moderate tooth sensitivity can occur in up to two‐thirds of patients during the early stages of the bleaching treatment. The sensitivity experienced during bleaching is believed to be due to changes in osmotic pressures. As the teeth are dehydrated during the bleaching procedures, a negative osmotic pressure develops that draws the odontoblastic processes into the dentinal tubules, which was explained by Brännström's hypersensitivity theory (Papathanasiou, Kastali, Perry, & Kugel, 2002). This also can explain why the risk of sensitivity is higher for use of bleaching lights or application of heat as these agents dehydrate the teeth and thus should be used with caution (Baik, Rueggeberg, & Liewehr, 2001; Buchalla & Attin, 2007).

Hydrogen peroxide bleaches teeth through a strong oxidizing action that breaks down pigments to molecules that are small enough to diffuse out of the tooth or small enough to absorb less light making the teeth appear lighter. Hydrogen peroxide forms free radicals such as oxygen and hydroxyl that penetrate through the enamel pores and into the dentin, which in turn breaks the carbon: carbon double bonds of the chromophore molecules on the teeth and degrade them to smaller and more stable molecules. The chroma concentration of chromatogens is reduced, hence the whitening effect (Dahl & Pallesen, 2003). Tooth sensitivity is also caused by the passage of the hydrogen peroxide molecules and free radicals through the enamel and dentin into the pulp because of their increased permeability, triggering pulpal inflammation, and stimulating pulpal sensory nerves (Haywood, 1992).

In an attempt to reduce the sensitivity and pain caused by tooth bleaching, various tooth sensitivity treatment options such as an application of fluoride, casein phosphopeptide‐amorphous calcium phosphate, and potassium nitrate were suggested. Potassium nitrate is one of the most common desensitizers in dentifrices. The potassium ions penetrate the length of the dentinal tubules and block repolarization of sensory nerve endings to decrease pain response (Kim, 1986). Fluoride and casein phosphopeptide‐amorphous calcium phosphate decrease sensitivity by tubule occlusion, thus preventing fluid flow (Zhao, Liu, Sun, & Zhang, 2011). Sensitivity during treatment may necessitate changing the bleaching product or the delivery system, shortening the duration of treatment, or prolonging the treatment interval.

Fluoride is widely used for not only prevention of caries but also treatment of hypersensitivity. NaF has been well known for its role in preventing demineralization, promoting remineralization of enamel, and increasing enamel hardness (Attin, 1997; Bizhang et al., 2006; Gürgan, Bolay, & Alaçam, 1997). One of the most common agents used for the prevention of tooth bleaching related sensitivity is also fluoride. Numerous studies (Bollineni et al., 2014; Haywood et al., 2005; Leonard, Smith, Garland, & Caplan, 2004; Marson, Sensi, Vieira, & Araújo, 2008; Singh, Ram, Shetty, Chand, & Yadav, 2010; Tay et al., 2009; Tschoppe, Neumann, Mueller, & Kielbassa, 2009) have been done on the effect of fluoride on bleaching related hypersensitivity. In a clinical study that used a fluoride containing desensitizing agent before an in‐office bleaching, hypersensitivity was reduced. The reduction in hypersensitivity might have been caused by fluoride increasing the enamel resistance to the demineralizing effect of the bleaching gel (Reis, Dalanhol, Cunha, Kossatz, & Loguercio, 2011).

The fluoride application after the bleaching may reverse the demineralizing effect on the enamel surface. The use of bleaching products containing fluoride resulted in fewer hazardous effects on enamel mineral content (Basting, Rodrigues, & Serra, 2003). The fluoride can also help in preventing future caries as the higher permeability of bleached enamel enhances the adhesion of cariogenic bacteria adhering to the enamel surface that can promote caries. A fluoridated bleaching gel resulted in less marked demineralization without affecting whitening efficacy (Chen, Chang, Liu, Chuang, & Yang, 2008). The addition of fluoride to the tooth whitening gel enhanced remineralization without affecting the gel's whitening efficacy (Gladwell, Simmons, & Wright, 2006). At this point, a fluoride containing abrasive paste seems to be favorable for tooth cleaning before in‐office bleaching.

In the present study, an artificial discolored bovine tooth model that was established in a previous study was used (Kishi, Otsuki, Sadr, Ikeda, & Tagami, 2011).

Tea extract was used as the staining medium similar to previous studies (Sulieman, Addy, MacDonald, & Rees, 2004; Sulieman, Addy, MacDonald, & Ress, 2005; Sulieman, Addy, & Rees, 2003), as the stains developed by tea is almost entirely organic and it simulates discoloration that develops in vivo. Stains produced by tea also have no potential for calcification, and the stains produced are easy to standardize, reproduce, and control (Sharif, MacDonald, Hughes, Newcombe, & Addy, 2000). In addition, tea is easily available, cheap, and simple to use.

According to the manufacturer's instruction of Shofu Hi‐Lite, consecutive bleaching procedure must be applied by three times at most in one visiting. Clinically, this in‐office bleaching treatment is repeated until acquiring the patient's satisfaction or by the maximal limitation of seven times. Between patient's visiting, bleached teeth are exposed to the saliva and have a possibility of remineralization. In the study design of this in vitro research, the effect of the saliva was eliminated. For the future study, the role of saliva should be considered. There are some methods to evaluate changes in tooth color. One is visual analysis by comparison with a standard shade guide, which is the most frequently applied method in clinical practice. Another method is the use of a colorimeter or a spectrophotometer (Watts a, 2001). Although the visual analysis using a shade guide is a simple, easy, and fast method to use, it is too subjective. The present study used a dental colorimeter (Shade Eye NCC), which was able to evaluate color changes objectively. It has been reported that the use of Shade Eye NCC for shade selection yields better results than the visual method within uncomplicated cases (Li & Wang, 2007). An advantage of a colorimeter is that it can generate exact values that can be easily analyzed statistically.

In this study, increasing the number of bleaching cycles enhanced the bleaching effect of Shofu Hi‐Lite regardless of the pretreatment method (Figure 3). The value increased per bleaching cycle and which were evident in the color photographs (Figure 2). The color difference (ΔE) between the experimental and control groups did not show any statistically significant difference (*p* > 0.05) as shown in Figure 4. This means that NaF did not interfere with the bleaching efficacy of 35% hydrogen peroxide contrary to the manufacturer's instructions. This result is in agreement with another study (Armênio et al., 2008) evaluating the use of a 1.23% fluoride gel in a bleaching tray after daily use of 16% carbamide peroxide gel. It was concluded that NaF did not prevent the peroxide gel from bleaching the tooth structures. A possible explanation for these results is that the trans‐enamel and trans‐dentinal diffusion of hydrogen peroxide is not affected by fluoride because of its low molecular weight. The peroxide molecule can pass easily through intact enamel and dentin in the interstitial space because of its minute size despite the tubule occluding action of fluoride (Tay et al., 2009). This shows that the tooth is a semipermeable membrane that is open to certain‐sized molecules (Cooper, Bokmeyer & Bowles, 1992). Despite these proven advantages of fluoride application prior to tooth bleaching, the manufacturer discourages this form of pretreatment, the reasons of which are somewhat unclear. However, the results of the current study run counter to the manufacturer's instructions and agree with the results of previous studies that fluoride application prior to bleaching does not interfere with bleaching efficacy. It is therefore suggested that for this particular product, a fluoride‐containing abrasive paste could be used prior to bleaching.

There are many factors concerning fluoride application affected on bleaching; kinds of fluoride, concentration, application method, and time. Further study is necessary to establish a suitable clinical method of fluoride application for prevention of tooth sensitivity at tooth bleaching.

## 5 CONCLUSION

Based on the results of this study, it can be concluded that the application of NaF before tooth bleaching did not interfere with the bleaching effect of the particular product tested.

### CLINICAL SIGNIFICANCE

Sodium fluoride can be applied before tooth bleaching procedure without altering bleaching properties.
